# Expression of Orf Virus B2L Protein and Monoclonal Antibody Preparation

**DOI:** 10.3390/microorganisms14081770

**Published:** 2026-08-12

**Authors:** Ying Liu, Jige Du, Fengye Han, Kaiying Zhu, Xin Guo

**Affiliations:** 1 State Key Laboratory of Veterinary Public Health Safety, College of Veterinary Medicine, China Agricultural University, Beijing 100193, China; liuying@cau.edu.cn; 2 China Institute of Veterinary Drug Control, Beijing 100081, China; du19371@163.com (J.D.); han1292656694@163.com (F.H.); zhukaiying224@163.com (K.Z.)

**Keywords:** orf virus, B2L, monoclonal antibody

## Abstract

Currently, there is no effective ELISA detection methods for orf virus (ORFV) based on recombinant proteins and monoclonal antibodies(mAbs). This research aims to express recombinant B2L protein (rB2L) of ORFV and prepare high specificity monoclonal antibodies targeting rB2L. After codon optimization for ORFV *B2L* gene, the optimized gene fragment was chemically synthesized and ligated into a prokaryotic pET-30a(+) vector for expression and purification of rB2L. Taking the purified rB2L as immunogen, three stable hybridoma cell lines (1F2, 2B3 and 3H6) secreting anti-ORFV mAbs were obtained through indirect ELISA combined with immunofluorescence. Subtype identification results indicated all three antibodies carried κ light chains with heavy chain subtypes IgG1, IgG2b and IgG2a, respectively. Indirect ELISA titer detection showed all three mAbs possessed titers higher than 1:6400. Among them, mAb 2B3 achieved the highest titer up to 1:25,600. Western blot verified that mAb 2B3 could specifically bind both rB2L and ORFV. Indirect immunofluorescence assay also confirmed mAb 2B3 could recognize viral antigens inside ORFV-infected primary goat testicular cells. These data suggest that rB2L and its specific monoclonal antibody 2B3 provide core experimental materials for developing an effective ORFV detection method.

## 1. Introduction

As a zoonotic virus, ORFV poses a constant threat to the development of the sheep industry and human health [1]. At present, ORFV infection is widely distributed, with outbreaks reported in Central Africa, the Middle East, Europe and Asia, severely endangering the healthy development of the sheep industry [2,3,4,5,6]. The morbidity in lambs can reach 100%, which seriously impairs feeding and growth, resulting in substantial economic losses to the livestock industry [7]. With the introduction and cross-regional transportation of sheep [8], the transmission and epidemic status of ORFV have become increasingly serious. Therefore, it is urgent to strengthen the detection, prevention and control of ORFV.

Currently, there is no specific drug available for ORFV infectionand vaccination remains the only preventive measure against this disease [9]. Although the development and application of ORFV-related vaccines have lasted for nearly a century and have played an important role in disease control, the commercially approved vaccines still present considerable safety or efficacy issues [7,10]. Furthermore, the lack of effective ORFV antigen/antibody detection kits makes it difficult to evaluate vaccine efficacy and screen negative animals. Hence, there is an urgent need to establish rapid, sensitive, and specific detection methods for ORFV antigens and antibodies. As the major antigenic protein of ORFV [8], establishing an ELISA assay for ORFV based on B2L and its monoclonal antibodies represents a promising strategy. In this study, we performed the generation, screening and application of anti-ORFV monoclonal antibodies using ORFV and its recombinant B2L protein.

## 2. Materials and Methods

### 2.1. CellsVirus, Plasmids, Animalsand Reagents

SP2/0 myeloma cell line and virus strain ORFV-CQ were preserved in our laboratory [11]. Six-week-old female BALB/c mice and multiparous BALB/c mice were procured from Beijing Vital River Laboratory Animal Technology Biotechnology (Beijing, China). All mice were euthanized by cervical dislocation at the end of the experiment. All animal immunization and antibody production procedures were approved by the Animal Care and Use Committee of the China Institute of Veterinary Drug Control. Conventional reagents for hybridoma cell fusion and molecular biology were used as described previously [12]. 

### 2.2. B2L Gene Amplification and Sequence Verification

Viral nucleic acid was extracted from clinical samples using the nucleic acid extraction kit according to the manufacturer’s instructions. Specific primers ORFV-*B2L*-F and R were designed based on the *B2L* gene sequence of orf virus isolate FX86 (GenBank accession No. PP943427.1) using Primer 5 software. The forward primer was ORFV-*B2L*-F: 5′-ATGTGGCCGTTCTCCTCCATC-3′ and the reverse primer was ORFV-*B2L*-R: 5′-TTAATTTATTGGCTTGCAGAACTC-3′. PCR was performed in a 25 μL reaction mixture containing 12.5 μL of 2 × PCR Master Mix, 1 μL of DNA template, 1 μL of each primer (10 pmol/μL) and nuclease-free-distilled water up to 25 μL. The amplification conditions were as follows: pre-denaturation at 95 °C for 3 min; 35 cycles of denaturation at 95 °C for 15 s, annealing at 56 °C for 30 s and extension at 72 °C for 2 min; a final extension at 72 °C for 10 min. PCR products were analyzed by 1.0% agarose gel electrophoresis and target bands were sent to Sangon Biotech (Shanghai) Co., Ltd. (Shanghai, China) for sequencing. The *B2L* sequence verified by sequencing served as the basis for subsequent codon optimization and artificial synthesis.

### 2.3. Plasmid Construction

The gene encoding rB2L with a C-terminal poly histidine (6 His) tag in this study was optimized following *Escherichia coli* expression system codon preferences and pET-G*B2L* was generated by standard recombinant DNA procedures as described previously [13].

### 2.4. Expression and Purification of Recombinant Proteins

The experimental scheme of protein expression and purification referred to the research method reported previously [13]. Briefly, pET-G*B2L* and pET-30a(+) were separately transformed into BL21(DE3) competent cells. IPTG was added to induce target protein expression under two different temperature conditions: 37 °C induction for 4 h and 15 °C for 16 h. After induction, SDS-PAGE was adopted to analyze the expression abundance and solubility of target protein under different induction parameters. Subsequently, rB2L was purified and stored at −80 °C.

### 2.5. Animal Immunization

Five female BALB/c mice were selected for immunization with rB2L. Each mouse received multipoint subcutaneous injection with a single immunization dosage of 50 μg rB2L. The immunization interval was set as 14 days with continuous three rounds of basic immunization. 7–10 days after the third injection, serum antibody titers of each mouse were detected via indirect ELISA. Mice with serum antibody titer higher than 1:12,800 were selected for booster immunization with 50 μg rB2L protein.The detailed immunization schedule is shown in Table 1.

### 2.6. Cell Fusion and Detection

Splenocytes obtained from immunized mice were fused with SP2/0 cells followed the protocol described previously [12]. Indirect ELISA and cell indirect immunofluorescence infected with ORFV-CQ were jointly used to screen hybridoma cell clones with specific positive signals. Positive clones were subjected to three rounds of limiting dilution subcloning to obtain stably hybridoma cell strains. The successfully screened cell lines were preserved in liquid nitrogen for long-term storage.

### 2.7. Monoclonal Antibody Subtype Identification

Cell culture supernatant of each hybridoma strain was collected and the heavy chain subtype and light chain type of secreted antibodies were tested strictly following the operating instructions of Biodragon monoclonal antibody subtype detection kit (Beijing, China).

### 2.8. Monoclonal Antibody Preparation

Multiparous female BALB/c mice were intraperitoneally injected with 0.5 mL Freund’s incomplete adjuvant for presensitization treatment. Ten days after adjuvant injection, about 1 × 10^6^ hybridoma cells were injected into each mouse. When the mice showed obvious abdominal distension and limited movement, ascites fluid was collected under sterile conditions. The total monoclonal antibodies in ascites were purified by Protein A affinity chromatography column (GE Healthcare, Chicago, IL, USA, 17-5079-01). Indirect ELISA was used to detect the antibody titer of purified mAbs followed the protocol described previously [12].

### 2.9. Western Blot Specificity Identification of Monoclonal Antibodies

Recombinant protein, virus particle lysate, protein and cell control sample were separated by SDS-PAGE and transferred onto a PVDF membrane under 50 V constant voltage for 2 h. The membrane was blocked with 5% skim milk solution for 90 min, then incubated with purified monoclonal antibody (dilution ratio 1:1500) at 37 °C for 1 h. After repeated washing with PBST buffer, HRP-labeled goat anti-mouse IgG secondary antibody (1:5000 dilution) was added and incubated at room temperature for 30 min. After final PBST cleaning, chemiluminescence imaging equipment was used to collect specific protein band signals.

### 2.10. Immunofluorescence (IFA) 

Primary goat testicular cells were inoculated with ORFV-CQ and cultured until typical cytopathic lesions appeared. Then cells were treated as described previously for IFA [12]. The diluted monoclonal antibody (1:200) was used as primary antibody and incubated with treated cells at 37 °C for 1 h. After three times of PBST washing, FITC-labeled rabbit anti-mouse IgG secondary antibody (1:500 dilution) was added and incubated for another 1 h at 37 °C. After repeated cleaning, the cell was placed under an inverted fluorescence microscope to observe green fluorescence signals.

## 3. Results

### 3.1. Amplification and Analysis of the B2L Gene

**Figure 1:**
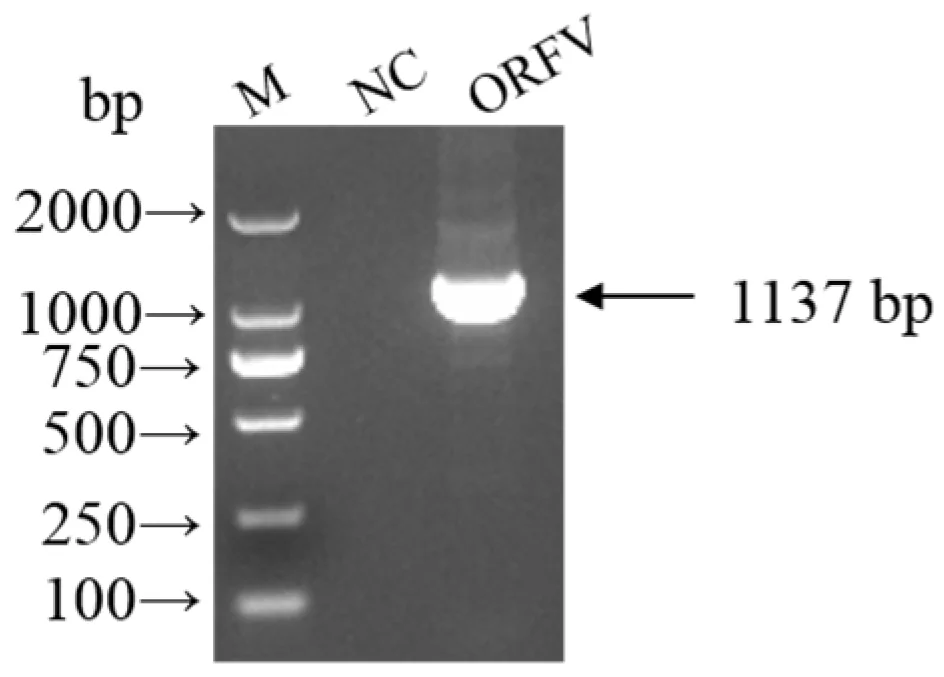
Amplification and identification of the *B2L* gene. M, DNA marker; NC, negative control.

Using nucleic acid extracted from ORFV-CQ as the template, PCR amplification was performed with primers ORFV-*B2L*-F and ORFV-*B2L*-R. A fragment of approximately 1000 bp was obtained, which was consistent with the expected size (1137 bp) (Figure 1). Gene sequencing and analysis confirmed that the amplified fragment was the *B2L* gene. The nucleotide sequence shared 97.78% identity with the *B2L* gene of the Orf virus isolate FX86 (Shaanxi, 2024). These results indicate that the Orf virus strain isolated in our laboratory (ORFV-CQ) is closely related to the prevalent strains circulating in China.

### 3.2. rB2L Was Efficiently Expressed in Escherichia coli

**Figure 2:**
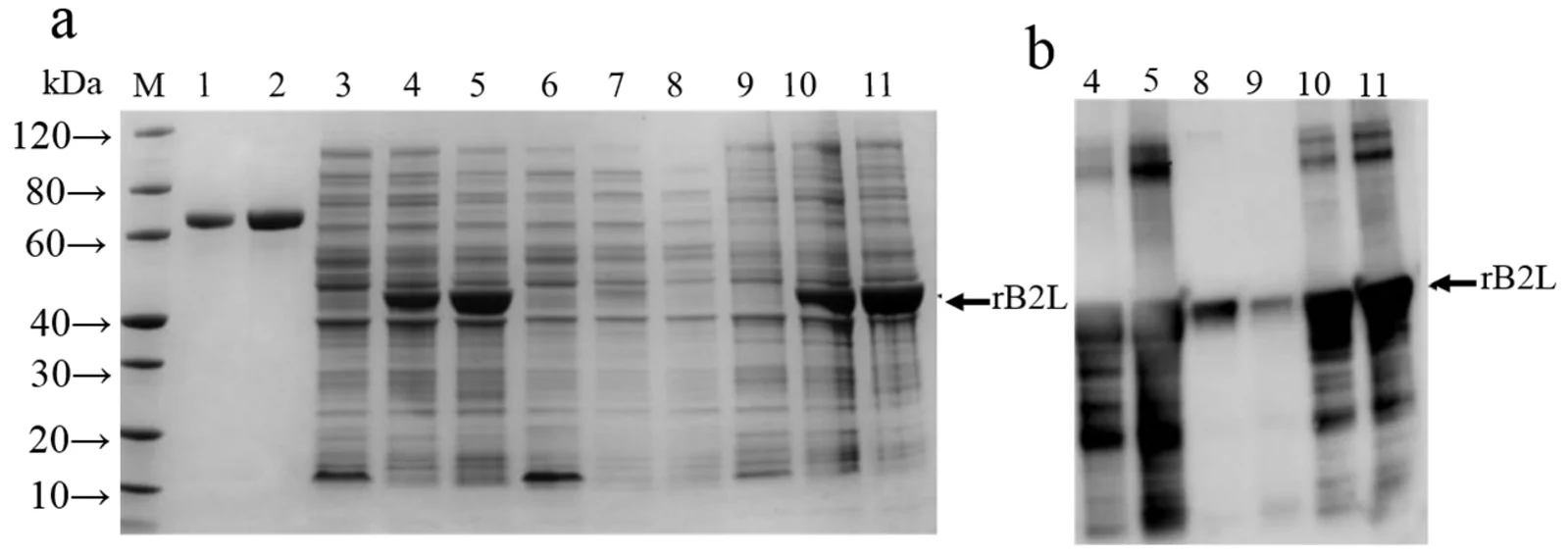
Identification of protein expression by SDS-PAGE and Western blot. (**a**) Identification of rB2L expression by SDS-PAGE; (**b**) identification of rB2L expression by Western blot with an anti-His monoclonal antibody. 1: BSA (1 μg). 2: BSA (2 μg). M: protein marker. 3: The cell lysates of pET induced with IPTG at 37 °C. 4: The cell lysates of pG*B2L* induced at 15 °C. 5: The cell lysates of pG*B2L* induced at 37 °C. 6: Supernatant of non-induced cell lysate. 7: Pellet of non-induced cell lysate. 8: The supernatant of cell lysates of pG*B2L* induced at 15 °C. 9: Pellet of cell lysate of pG*B2L* induced at 15 °C. 10: The supernatant of cell lysates of pG*B2L* induced at 37 °C. 11: Pellet of cell lysate of pG*B2L* induced at 37 °C.

The ORFV-CQ *B2L* gene was synthesized and cloned into a prokaryotic expression vector to obtain the recombinant expression plasmids pET30a-G*B2L* (referred to as pG*B2L*). *E. coli* BL21 (DE3) cells transformed with recombinant plasmid were cultured and induced with IPTG. SDS-PAGE analysis revealed that 6X histidine tagged rB2L was expressed predominantly in the form of inclusion bodies under both 15 °C and 37 °C induction conditions with a molecular weight between 40 and 50 kDa (Figure 2a), consistent with the expected size (42.7 kDa), which was confirmed by western blot analysis using antibody against His (Figure 2b). Expression of rB2L was lower at 15 °C, while expression level was higher at 37 °C. Therefore, the form of inclusion bodies induced at 37 °C was selected for purification.

### 3.3. Purification of rB2L

**Figure 3:**
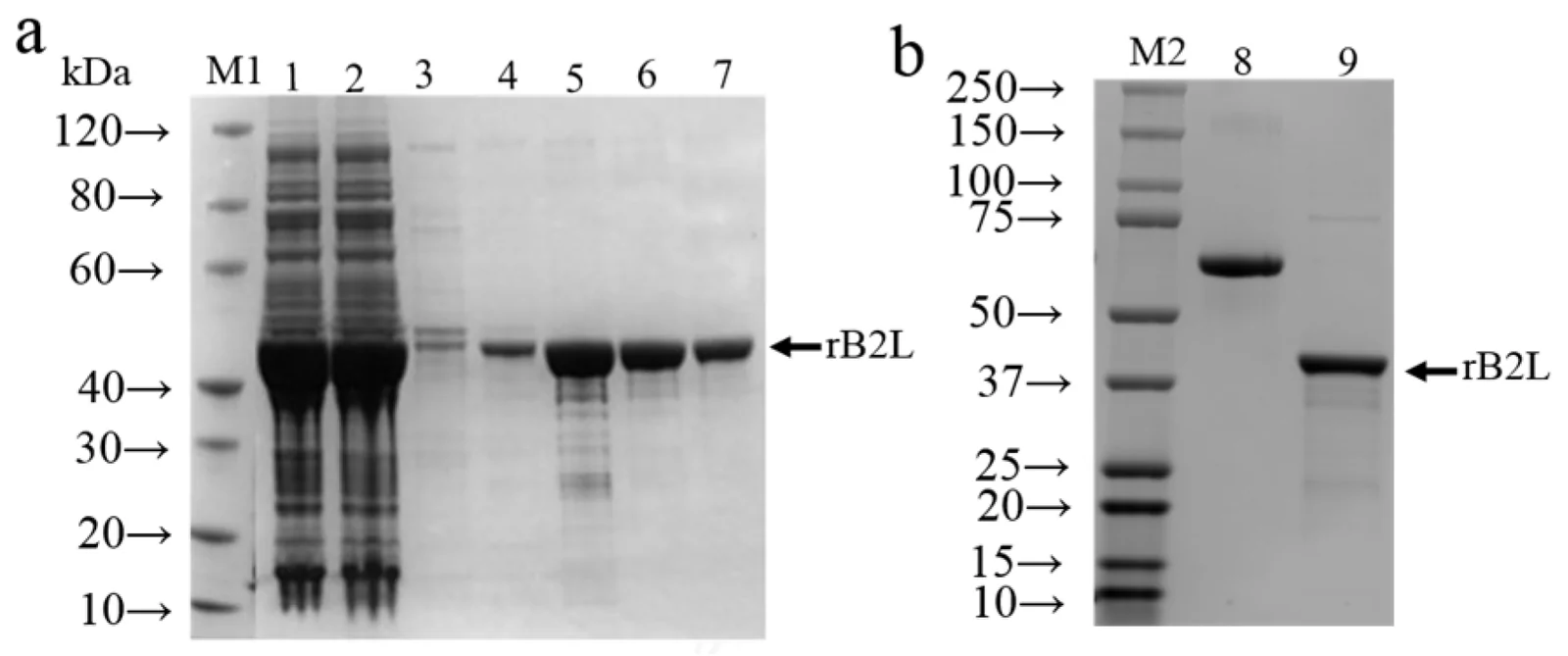
(**a**) Purification of recombinant protein. (**b**) Identification of purified protein by SDS-PAGE. M1, M2: protein marker. 1: The supernatant of dissolved inclusion bodies after centrifugation. 2: The flow-through from Ni-IDA resin after incubation with the supernatant. 3: The elution from Ni-IDA resin washed with elution buffer (containing 20 mM imidazole). 4: The elution from Ni-IDA resin washed with elution buffer (containing 50 mM imidazole). 5–6: The elution from Ni-IDA resin washed with elution buffer (containing 500 mM imidazole). 7: The elution from Ni-IDA resin. 8: BSA. 9: The recombinant protein purified.

rB2L eluted with buffer containing 500 mM imidazole (Figure 3a, lane 5–6) were collected throughout the purification process to obtain the purified recombinant protein. The measured concentration of purified rB2L protein was 1.02 mg/mL and the purity of the final protein product exceeded 92% (Figure 3b), which could meet the dosage requirements of subsequent animal immunization and antibody detection experiments.

### 3.4. Positive Hybridoma Cell Clones Were Screened by Indirect ELISA and IFA 

After cell fusion, rB2L-coated indirect ELISA and ORFV-infected cell immunofluorescence were used for double screening of positive hybridoma clones. After three rounds of limiting dilution subcloning, three hybridoma strains with stable secretion of anti-ORFV B2L monoclonal antibodies were successfully obtained, named as 1F2, 2B3 and 3H6, respectively. Antibody subtype identification test results showed that the light chains of the three mAbs all belonged to κ type and the heavy chain subtypes of 1F2, 2B3 and 3H6 were IgG1, IgG2b and IgG2a, respectively.

### 3.5. High ELISA Titer Was Observed for the Purified Monoclonal Antibody

**Figure 4:**
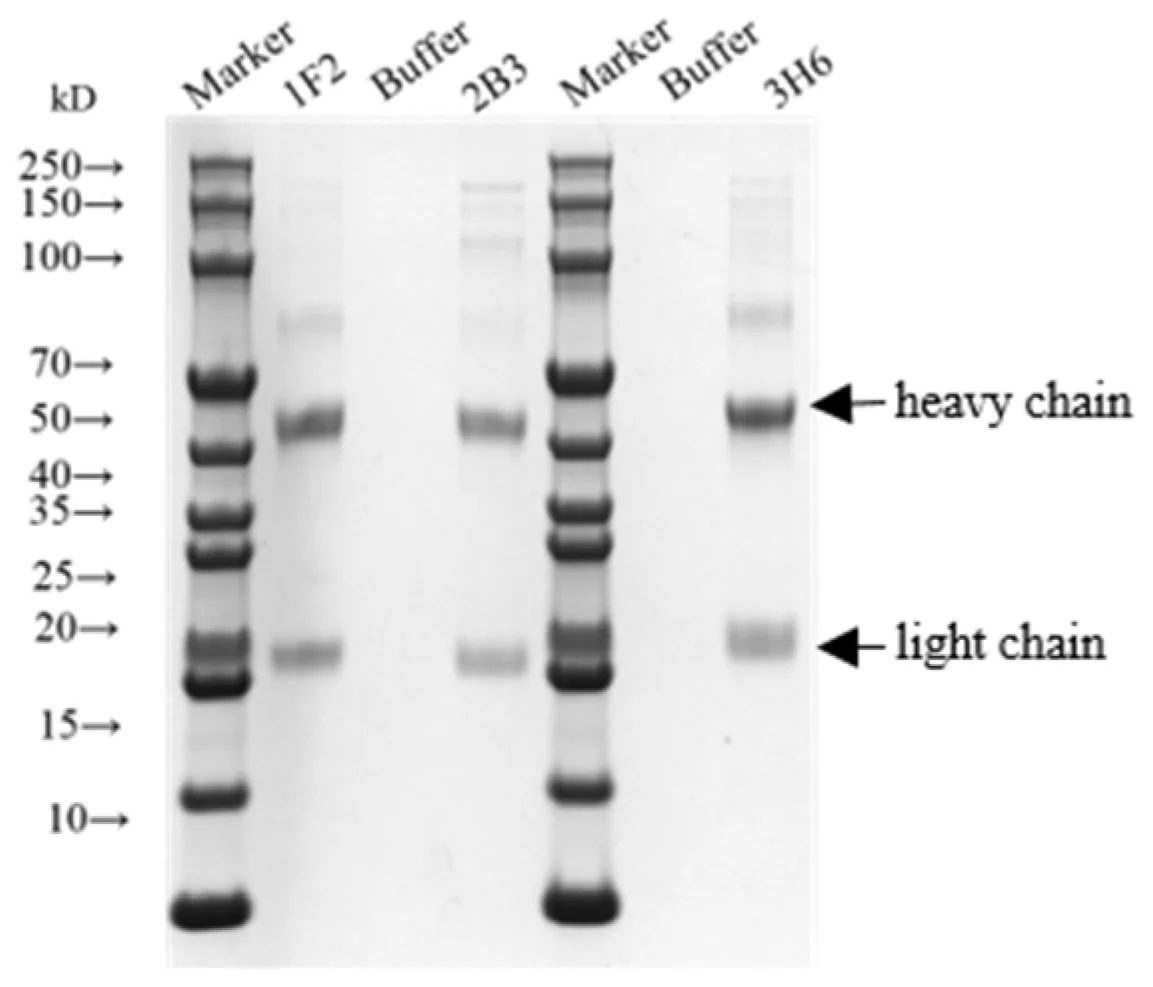
Identification of monoclonal antibodies by SDS-PAGE.

Three hybridoma strains were expanded for ascites preparation and ascites of mAbs were purified. SDS-PAGE identification showed the purity of all three antibodies was higher than 90% (Figure 4). The measured concentrations of purified 1F2, 2B3 and 3H6 mAbs were 2.0 mg/mL, 1.9 mg/mL and 1.3 mg/mL, respectively. Indirect ELISA detection results revealed none of the three antibodies produced specific positive signals with irrelevant His-tagged protein rCSA-His, indicating no cross-reaction with His label. The ELISA titers of all three mAbs against rB2L were higher than 1:6400. Among them, mAb 2B3 showed the highest sensitivity with a titer reaching 1:25,600. Therefore, mAb 2B3 was selected for subsequent specificity verification tests.

### 3.6. Western Blot Identification of mAb 2B3

**Figure 5:**
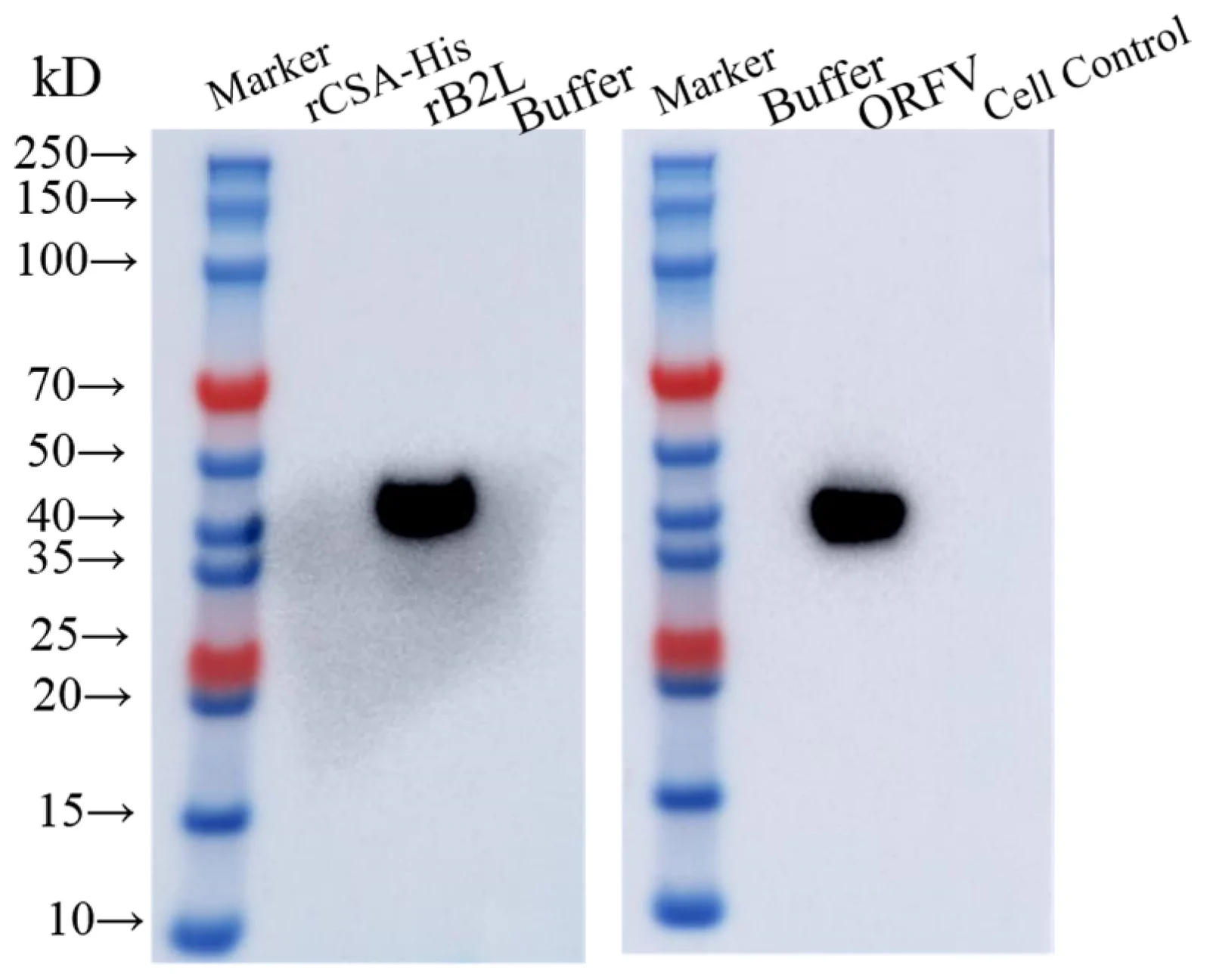
Identification of mAb 2B3 by Western blot.

Western blot experiment was designed to evaluate the binding specificity of mAb 2B3 with rB2L and ORFV-CQ. The results showed obvious single specific band appeared at the position of 40–50 kDa in both rB2L and ORFV virus groups, which matched the theoretical molecular weight of 42.7 and 42.6 kDa, respectively. No specific band signal was observed in the irrelevant protein rCSA-His and blank cell control groups (Figure 5), proving mAb 2B3 can specifically bind both rB2L and native viral B2L antigen.

### 3.7. Indirect Immunofluorescence Identification of mAb 2B3

**Figure 6:**
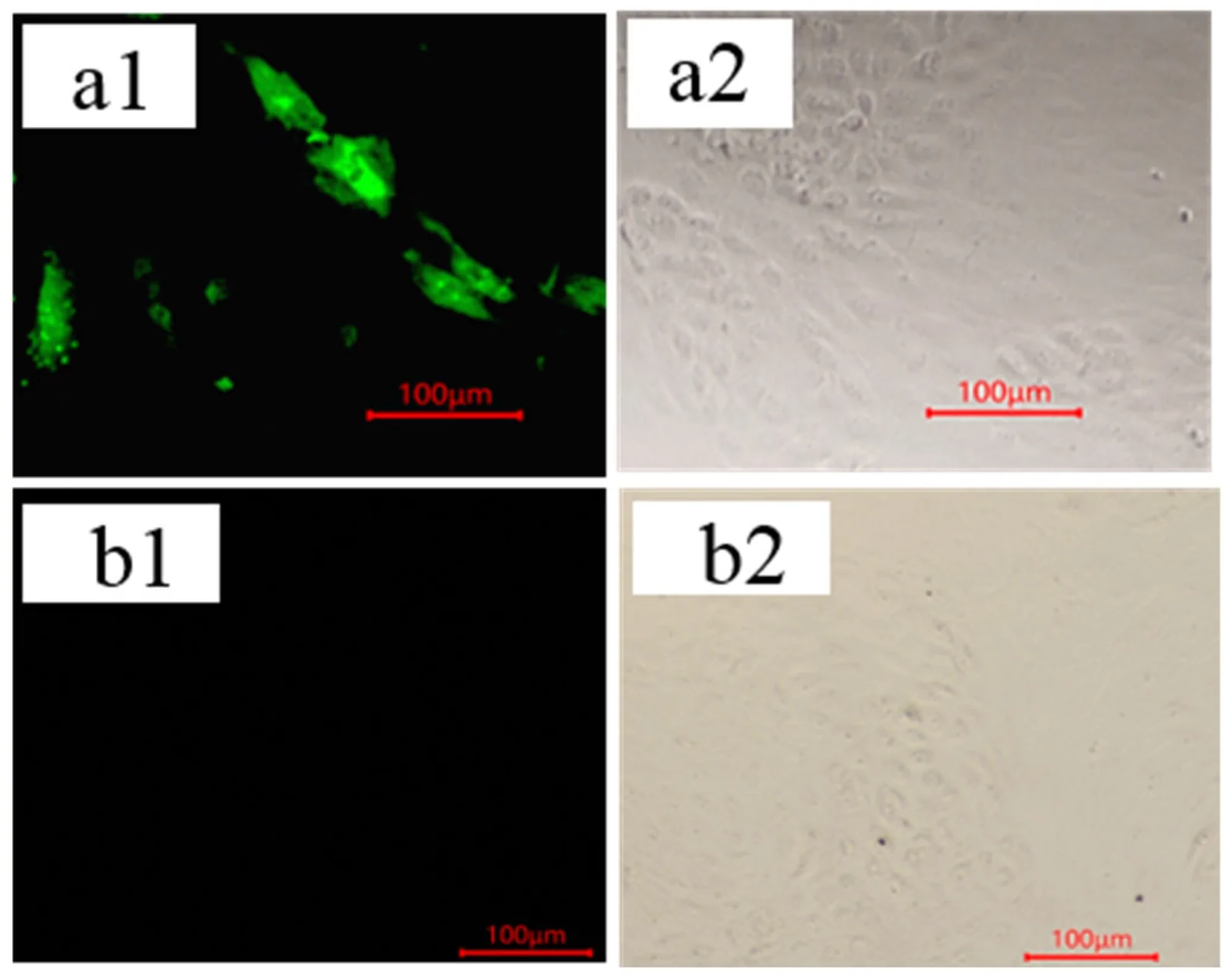
Specific interaction between monoclonal antibody 2B3 and ORFV identified by indirect immunofluorescence. Primary Goat Testicular cells infected with ORFV-CQ. (**a1**) Fluorescence image; (**a2**) bright-field image. Cell control. (**b1**) Fluorescence image; (**b2**) bright-field image.

Primary goat testicular cells infected with ORFV-CQ were taken as detection samples and uninfected normal cells were set as blank control. Indirect immunofluorescence results showed strong specific green fluorescence signals in ORFV-infected cell lesions when mAb 2B3 was used as primary antibody and no obvious non-specific green fluorescence was observed in the uninfected blank cell group (Figure 6). This result verified that mAb 2B3 can recognize natural conformational epitopes of B2L protein expressed in ORFV-infected cells.

## 4. Discussion

ORFV is the causative agent of contagious ecthyma (orf) in sheep and can infect ruminants such as sheep and goats as well as humans, causing severe economic losses to the global sheep industry [8]. As a core functional gene of ORFV, the B2L encodes the major immunogenic envelope protein B2L of the virus [14,15]. This protein is highly conserved and strongly immunogenic, serving as a key target for the diagnosis of ORFV infection, vaccine development, and research on pathogenic mechanisms [16,17,18,19]. The preparation of monoclonal antibodies (mAbs) is fundamental for establishing high quality antigens and detection methods. Previous studies have demonstrated that the antigenic epitopes of B2L are dominated by conformational epitopes, whose integrity depends on the native spatial structure of the protein and cannot be recognized by antibodies under denaturing conditions (e.g., Western blot assay) [18]. The selection of virus-like particles that retain the native conformation of the antigen or B2L protein with favorable conformational epitopes is critical for the preparation of its monoclonal antibodies. In this study, the *B2L* gene of ORFV-CQ was optimized and artificially synthesized according to the codon preference of *Escherichia coli* and was successfully expressed in the prokaryotic expression system, with a yield of up to 45 mg/L at 37 °C. Although the proportion of soluble expression was low, the recombinant B2L protein obtained after inclusion body purification exhibited high concentration and purity, laying a material foundation for the subsequent establishment of a B2L-based ELISA method.

The recombinant B2L used in this study lacks post-translational modifications provided by eukaryotic expression systems. If only rB2L was used as immunogen and the coating antigen for screening in indirect ELISA, it would be highly likely to obtain monoclonal antibodies that only target rB2L but fail to react with the native ORFV. Therefore, in the screening process of monoclonal antibodies, this study adopted both indirect ELISA with B2L as the coating antigen and indirect immunofluorescence assay (IFA) using ORFV-infected cells. The combined use of indirect ELISA and IFA for screening anti-ORFV monoclonal antibodies not only maximally ensures the specificity of the mAbs, but also guarantees the convenience and repeatability of the coating antigen. In the indirect ELISA experiments, recombinant Clostridium septicum α toxin with a His-tag was selected as the control to eliminate interference from the His-tag, confirming that all three monoclonal antibodies could react with rB2L. Based on the comprehensive analysis of the concentration, subtype, and indirect ELISA titer of the purified monoclonal antibodies, mAb 2B3 was selected for subsequent Western blot and indirect immunofluorescence assays. Compared with mAb 2E4, which did not recognize epitopes of B2L according to Western blotting and cannot be used for detection under denaturing conditions [18], mAb 2B3 obtained in this study exhibits a wider range of applications. The purified mAb 2B3 can recognize not only native ORFV (positive in IFA), but also denatured ORFV and rB2L (Western blot), offering broader application scenarios. The rB2L used in this study lacks eukaryotic post-translational modifications. Although broad-spectrum mAbs were successfully prepared, subsequent validation of their reactivity with different ORFV genotypes is still needed. To facilitate the better application of mAb 2B3 in serological detection methods for ORFV antigens and antibodies, further research is required to identify the antigenic epitope information targeted by this monoclonal antibody.

## Figures and Tables

**Table 1:** Immunization procedures for mice.

Number of Immunizations	Immunization Route	Immunization Dose (μg per Mouse)
The first immunization	Multipoint subcutaneous injections in the back and neck	50 μg
The second immunization	Multipoint subcutaneous injections in the back and neck	50 μg
The third immunization	Multipoint subcutaneous injections in the back and neck	50 μg
Strengthen immunization	Intraperitoneal injection (recombinant protein)	50 μg

## Data Availability

The original contributions presented in the study are included in the article. Further inquiries can be directed to the corresponding author.
